# Submucosal injection–related foreign body reaction complicating endoscopic submucosal dissection: this time, it is not the usual suspect

**DOI:** 10.1016/j.igie.2026.02.007

**Published:** 2026-02-14

**Authors:** Fouad Jaber, Rollin George, Roshan Raza, Salmaan Jawaid

**Affiliations:** 1 Department of Medicine, Section of Gastroenterology and Hepatology, Baylor College of Medicine, Houston, Texas, USA; 2 Department of Clinical Pathology, Baylor College of Medicine, Houston, Texas, USA

Although submucosal injection of a lifting agent is a key step during endoscopic resection of colon polyps, its impact is not completely benign. Foreign body giant cell reactions have been well described with ORISE Gel (Boston Scientific, Marlborough, Mass, USA), complicating both resection and histopathologic interpretation.^1^^,^^2^ Reports with newer agents remain limited. We describe a case of giant cell reaction after EverLift (Laborie Medical Technologies Corp, Portsmouth, NH, USA) injection.

A 69-year-old female underwent surveillance colonoscopy, revealing a 10-mm flat polyp in the ascending colon with Japan Narrow-Band Imaging Expert Team 2A and 2B features (Fig. 1A and B). Given the lesion's flat morphology, right-sided location, and optical features raising concern for submucosal fibrosis or early invasive characteristics, submucosal injection was performed. Submucosal injection with 3 mL of EverLift failed to achieve adequate lift, and thus, resection was deferred. After biopsy results showed a tubular adenoma, she was referred for endoscopic submucosal dissection (ESD). During ESD, an unusual-appearing tissue was observed within the center of the resection bed (Fig. 1C), adherent to the muscularis propria, raising concern for hypertrophied muscle or deep submucosal invasion. En bloc resection was achieved with some difficulty. Histopathology revealed a tubular adenoma with R0 resection along with amorphous hyaline-like material with a surrounding foreign body giant cell reaction (Fig. 1D).

**Figure 1:**
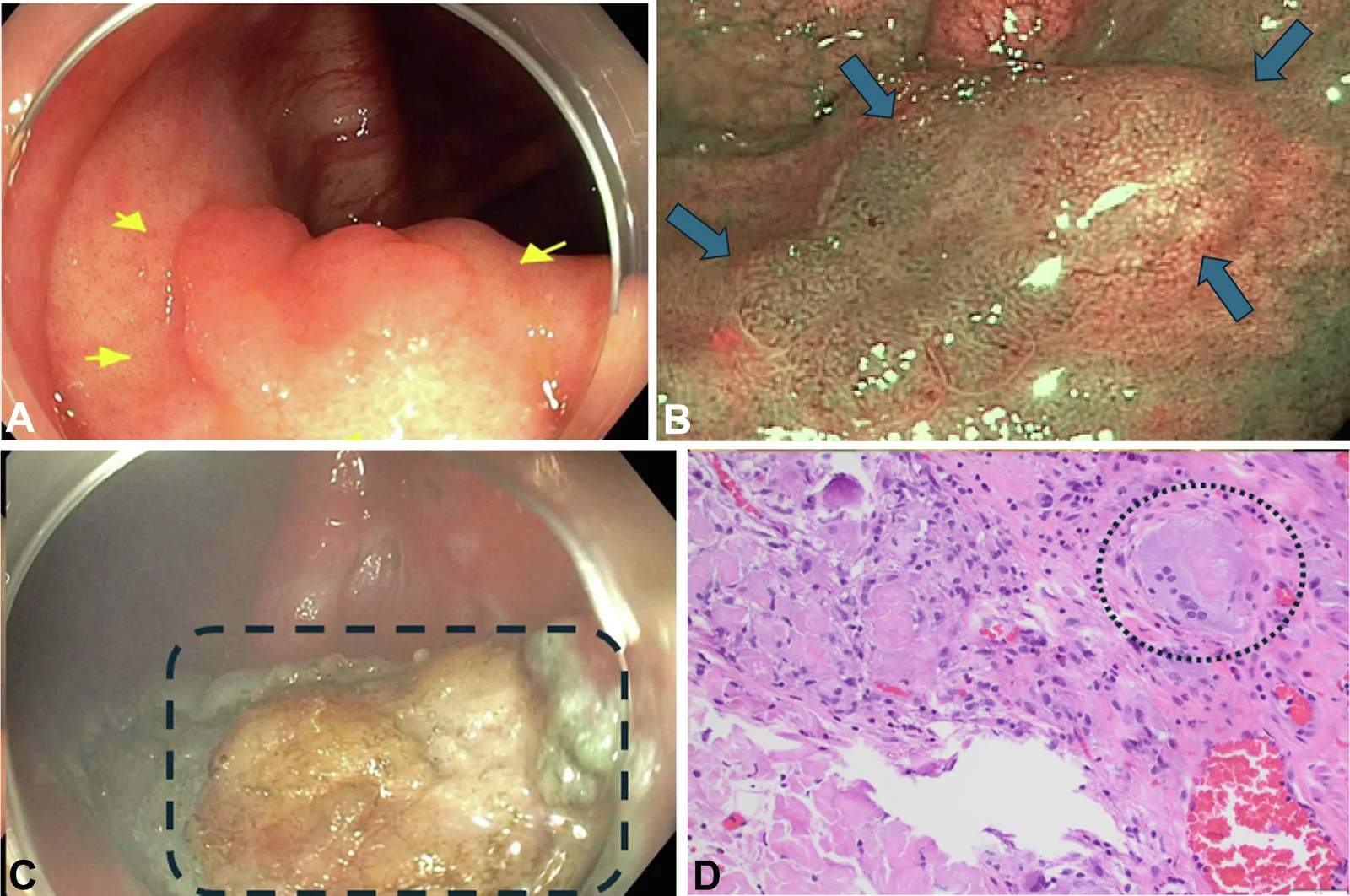
**A,** A 10-mm sessile polyp in the ascending colon (*yellow arrows*). **B,** The polyp under narrow-band imaging, demonstrating mixed Japan Narrow-Band Imaging Expert Team 2A and 2B features (*blue arrows*). **C,** After resection, unusual tissue was noted within the center of the resection bed, adherent to the muscularis propria (*dotted square*). **D,** Hematoxylin and eosin, original magnification ×100, showed amorphous hyaline material and debris with associated giant cell reaction (*dotted circle*).

Traditionally, normal saline solution—often mixed with methylene blue—has been used for submucosal lifting but is rapidly absorbed, frequently necessitating reinjection. Other short-acting solutions, including glycerol, hyaluronic acid, hydroxypropyl methylcellulose, and hydroxyethyl starch, have also been used and have not been associated with foreign body reactions.^3^ More recently, U.S. Food and Drug Administration–approved synthetic lifting agents such as Eleview (Medtronic, Minneapolis, Minn, USA) (2017)^4^ and ORISE (2018)^5^ have gained widespread adoption due to their durable submucosal cushion and potential to reduce procedure time. However, foreign body giant cell reactions have been most commonly reported with synthetic agents, particularly ORISE gel,^1^^,^^2^ which was later removed from the market after reports of persistent submucosal reactions that raised concern for malignancy and resulted in unnecessary surgical resections. Such reactions have been reported less frequently with other polymer-based formulations. Although EverLift has been designed to improve lift durability and procedural efficiency, data regarding its long-term tissue interactions remain limited.

Our case adds to the growing body of evidence that synthetic lifting agents, including newer formulations, may not be entirely inert and can elicit foreign body reactions. To the best of our knowledge, this represents the first reported case of a foreign body reaction associated with EverLift. Recognition of this benign reactive process is critical to prevent overdiagnosis of malignancy or invasive disease.

## Patient Consent

The patient in this article has given written informed consent to publication of their case details.

## Disclosure

All authors disclosed no financial relationships.
